# Supplementary material for: Estimates of functional muscle strength from a novel progressive lateral step-up test are feasible, reliable, and related to physical activity in children with cerebral palsy
Source: PLoS One. 2024 Jul 10;19(7):e0306529. doi: 10.1371/journal.pone.0306529 (PMC11236174; doi:10.1371/journal.pone.0306529)
Supplement: S1 Table — (DOCX) [file pone.0306529.s001.docx]

S1 Table. Linear regression predicting physical activity counts at the ankle and hip in children with cerebral palsy using lateral step-up (LSUT) performance.

| Measure | Coefficients | β | t-value | SE | *p* | Std β | 95 % CI | Model R^2^, adj R^2^ |
| --- | --- | --- | --- | --- | --- | --- | --- | --- |
| Ankle (cts/day) |  |  |  |  |  |  |  | 0.204, 0.185 |
|  | Intercept | 911951 | 6.479 | 140758 | <0.001 |  | 628086 to 1195817 |  |
|  | LSUT 10 cm | 47116 | 3.320 | 14193 | 0.002 | 0.452 | 18493 to 75739 |  |
|  |  |  |  |  |  |  |  | 0.188, 0.169 |
|  | Intercept | 1010735 | 8.350 | 121051 | <0.001 |  | 766612 to 1254858 |  |
|  | LSUT 15 cm | 40358 | 3.151 | 12808 | 0.003 | 0.433 | 14528 to 66187 |  |
|  |  |  |  |  |  |  |  | 0.221, 0.203† |
|  | Intercept | 993035 | 8.492 | 116936 | <0.001 |  | 757210 to 1228860 |  |
|  | LSUT 20 cm | 45993 | 3.493 | 13169 | 0.001 | 0.470 | 19436 to 72551 |  |
|  |  |  |  |  |  |  |  | 0.213, 0,194† |
|  | Intercept | 966996 | 7.744 | 124864 | <0.001 |  | 715183 to 1218809 |  |
|  | LSUT_composite_ | 10244 | 3.407 | 3007 | 0.001 | 0.461 | 4180 to 16308 |  |
| Hip (cts/day) |  |  |  |  |  |  |  | 0.071, 0.050 |
|  | Intercept | 654518 | 7.684 | 85176 | <0.001 |  | 482745 to 826291 |  |
|  | LSUT 10 cm | 15592 | 1.816 | 8588 | 0.076 | 0.267 | -1728 to 32913 |  |
|  |  |  |  |  |  |  |  | 0.082, 0.061 |
|  | Intercept | 675610 | 9.373 | 72081 | <0.001 |  | 530244 to 820975 |  |
|  | LSUT 15 cm | 14954 | 1.961 | 7627 | 0.056 | 0.286 | -426.4 to 30335 |  |
|  |  |  |  |  |  |  |  | 0.111, 0.091 |
|  | Intercept | 660636 | 9.442 | 69965 | <0.001 |  | 519538 to 801735 |  |
|  | LSUT 20 cm | 18288 | 2.321 | 7879 | 0.025 | 0.334 | 2399 to 34178 |  |
|  |  |  |  |  |  |  |  | 0.094, 0.072 |
|  | Intercept | 659044 | 8.782 | 75049 | <0.001 |  | 507694 to 810393 |  |
|  | LSUT_composite_ | 3807 | 2.106 | 1807 | 0.041 | 0.306 | 162 to 7451 |  |

Physical activity in counts/day (cts/day); LSUT 10 cm, 15 cm, and 20 cm = repetitions at 10, 15, and 20 cm step heights, respectively; †*p* < 0.001
