# Supplementary material for: Estimates of functional muscle strength from a novel progressive lateral step-up test are feasible, reliable, and related to physical activity in children with cerebral palsy
Source: PLoS One. 2024 Jul 10;19(7):e0306529. doi: 10.1371/journal.pone.0306529 (PMC11236174; doi:10.1371/journal.pone.0306529)
Supplement: S2 Table — (DOCX) [file pone.0306529.s002.docx]

S2 Table. Linear regression predicting physical activity counts at the ankle and hip in typically developing control children using lateral step-up (LSUT) performance.

| Measure | Coefficients | β | t-value | SE | *p* | Std β | 95% CI | Model R^2^, adj R^2^ |
| --- | --- | --- | --- | --- | --- | --- | --- | --- |
| Ankle (cts/day) |  |  |  |  |  |  |  | 0.027, 0.005 |
|  | Intercept | 1688254 | 4.954 | 340817 | <0.001 |  | 1000931 to 2375578 |  |
|  | LSUT 10 cm | 18140 | 1.099 | 16512 | 0.278 | 0.165 | -15159 to 51440 |  |
|  |  |  |  |  |  |  |  | 0.008, -0.015 |
|  | Intercept | 1866021 | 5.628 | 331588 | <0.001 |  | 1197309 to 2534733 |  |
|  | LSUT 15 cm | 9553 | 0.580 | 16468 | 0.565 | 0.088 | -23658 to 42765 |  |
|  |  |  |  |  |  |  |  | 0.016, -0.007 |
|  | Intercept | 1823113 | 6.363 | 286536 | <0.001 |  | 1245257 to 2400970 |  |
|  | LSUT 20 cm | 12740 | 0.833 | 15288 | 0.409 | 0.126 | -18091 to 43570 |  |
|  |  |  |  |  |  |  |  | 0.019, -0.004 |
|  | Intercept | 1767452 | 5.469 | 323169 | <0.001 |  | 1115720 to 2419183 |  |
|  | LSUT_composite_ | 3350 | 0.910 | 3681 | 0.368 | 0.137 | -4073 to 10773 |  |
| Hip (cts/day) |  |  |  |  |  |  |  | 0.018, -0.005 |
|  | Intercept | 846184 | 4.413 | 191766 | <0.001 |  | 459450 to 1232917 |  |
|  | LSUT 10 cm | 8191 | 0.882 | 9291 | 0.383 | 0.133 | -10545 to 26928 |  |
|  |  |  |  |  |  |  |  | <0.001, -0.023 |
|  | Intercept | 1009083 | 5.414 | 186390 | <0.001 |  | 633192 to 1384974 |  |
|  | LSUT 15 cm | 92.97 | 0.010 | 9257 | 0.992 | 0.002 | -18575 to 18761 |  |
|  |  |  |  |  |  |  |  | <0.001, -0.023 |
|  | Intercept | 1005677 | 6.218 | 161728 | <0.001 |  | 679523 to 1331832 |  |
|  | LSUT 20 cm | 289.5 | 0.034 | 8629 | 0.973 | 0.005 | -17112 to 17691 |  |
|  |  |  |  |  |  |  |  | 0.003, -0.021 |
|  | Intercept | 951570 | 5.216 | 182447 | <0.001 |  | 583631 to 1319509 |  |
|  | LSUT_composite_ | 695.9 | 0.335 | 2078 | 0.739 | 0.051 | -3495 to 4887 |  |

Physical activity in counts/day (cts/day); LSUT 10 cm, 15 cm, and 20 cm = repetitions at 10, 15, and 20 cm step heights, respectively
