# Supplementary material for: Estimates of functional muscle strength from a novel progressive lateral step-up test are feasible, reliable, and related to physical activity in children with cerebral palsy
Source: PLoS One. 2024 Jul 10;19(7):e0306529. doi: 10.1371/journal.pone.0306529 (PMC11236174; doi:10.1371/journal.pone.0306529)
Supplement: Attachment — Submitted filename: Review comments on Manuscript Number.doc [file pone.0306529.s003.doc]

**Review comments on Manuscript Number: PONE-D-23-23848. Entitled “Estimates of functional muscle strength from a novel progressive lateral step-up test are feasible, reliable, and related to physical activity in children with cerebral palsy"**

**Overall, the idea of research is very interesting to be studied nowadays and paper is coherently developed. However, there are some comments and suggestions.**

**Title**

- Well structured

**Abstract**

- It is recommended to write 5 to 7 keywords in alphabetical order.

**Introduction**

- Well structured

**Subjects and methods**

- The authors recruited ambulant children with CP. However, there is great difference between unilateral and bilateral CP cases regarding energy expenditure, balance , strength. Authors should clarify which subtype of CP was included in their study.

**Statistical analysis**

- Well structured

**Discussion**

- Well structured

**References**

Update your References
